# Assessing tumor vascularization as a potential biomarker of imatinib resistance in gastrointestinal stromal tumors by dynamic contrast-enhanced magnetic resonance imaging

**DOI:** 10.1007/s10120-016-0672-7

**Published:** 2016-12-19

**Authors:** Lorena Consolino, Dario Livio Longo, Marianna Sciortino, Walter Dastrù, Sara Cabodi, Giovanni Battista Giovenzana, Silvio Aime

**Affiliations:** 1 Department of Molecular Biotechnology and Health Sciences, University of Torino, Via Nizza 52, 10126 Turin, Italy; 2 CAGE Chemicals srl, Via Bovio 6, 28100 Novara, Italy; 3 Institute of Biostructure and Bioimaging, National Research Council of Italy (CNR) c/o Molecular Biotechnologies Center, Via Nizza 52, 10126 Turin, Italy; 4 Department of Pharmaceutical Sciences, University of Eastern Piedmont, Largo Donegani 2/3, 28100 Novara, Italy

**Keywords:** Gastrointestinal stromal tumor, Angiogenesis, DCE-MRI, Imatinib, Gadolinium contrast agent

## Abstract

**Background:**

Most metastatic gastrointestinal stromal tumors (GISTs) develop resistance to the first-line imatinib treatment. Recently, increased vessel density and angiogenic markers were reported in GISTs with a poor prognosis, suggesting that angiogenesis is implicated in GIST tumor progression and resistance. The purpose of this study was to investigate the relationship between tumor vasculature and imatinib resistance in different GIST mouse models using a noninvasive magnetic resonance imaging (MRI) functional approach.

**Methods:**

Immunodeficient mice (*n* = 8 for each cell line) were grafted with imatinib-sensitive (GIST882 and GIST-T1) and imatinib-resistant (GIST430) human cell lines. Dynamic contrast-enhanced MRI (DCE-MRI) was performed on GIST xenografts to quantify tumor vessel permeability (*K* ^trans^) and vascular volume fraction (*v*_p_). Microvessel density (MVD), permeability (mean dextran density, MDD), and angiogenic markers were evaluated by immunofluorescence and western blot assays.

**Results:**

Dynamic contrast-enhanced magnetic resonance imaging showed significantly increased vessel density (*P* < 0.0001) and permeability (*P* = 0.0002) in imatinib-resistant tumors compared to imatinib-sensitive ones. Strong positive correlations were observed between MRI estimates, *K* ^trans^ and *v*_p_, and their related ex vivo values, MVD (*r* = 0.78 for *K* ^trans^ and *r* = 0.82 for *v*_p_) and MDD (*r* = 0.77 for *K* ^trans^ and *r* = 0.94 for *v*_p_). In addition, higher expression of vascular endothelial growth factor receptors (VEGFR2 and VEFGR3) was seen in GIST430.

**Conclusions:**

Dynamic contrast-enhanced magnetic resonance imaging highlighted marked differences in tumor vasculature and microenvironment properties between imatinib-resistant and imatinib-sensitive GISTs, as also confirmed by ex vivo assays. These results provide new insights into the role that DCE-MRI could play in GIST characterization and response to GIST treatment. Validation studies are needed to confirm these findings.

**Electronic supplementary material:**

The online version of this article (doi:10.1007/s10120-016-0672-7) contains supplementary material, which is available to authorized users.

## Introduction

Gastrointestinal stromal tumor (GIST) is the most common malignant mesenchymal neoplasm of the digestive tract, with a mean annual incidence of 11–14 patients per million people. Surgical resection is the first-line treatment for localized or resectable GISTs. However, every GIST is considered to be potentially malignant, and metastases are observed in liver or the peritoneal cavity in 50% of cases following primary surgical resection [1, 2]. GISTs are commonly distinguished from other sarcomas by gain-of-function mutations of the tyrosine kinase KIT receptor [3]. Imatinib (Gleevec; Novartis Pharmaceuticals) is a potent inhibitor of KIT and is currently the only effective treatment against metastatic and unresectable GIST [4–7]. However, clinical data show that imatinib fails to completely eradicate the disease, since most patients develop resistance after a few months of treatment, with significant complications observed in follow-up studies [8, 9].

The evaluation of GIST prognosis along the transformation from benign to malignant tumor is currently based on the Fletcher classification system that allows easy and accurate stratification of GIST patients according to tumor size, mitotic count, and anatomic location [10]. Moreover, recent studies have demonstrated the prognostic significance of some molecular markers such as the proliferating cell nuclear antigen Ki-67 and KIT mutational status [11, 12]. However, most of these GIST signatures can only be evaluated after surgical resection of the whole tumor, or the evaluation may be biased by the limited sampling associated with biopsies, thus hindering the prognosis for inoperable GISTs.

Notably, several ex vivo investigations have reported associations between vascularization and angiogenic markers in GIST cases with the poorest prognoses [13–16]. Considering the prognostic role of angiogenesis in GIST, the identification of reliable noninvasive tools that are able to monitor tumor vascularization may provide new insights into GIST characterization and therapy response evaluation. Solid tumors typically display altered and unstructured vasculature that is responsible for irregular perfusion and permeability [17], and these properties can be assessed using several imaging approaches that allow the visualization of intratumoral vessels [18, 19]. Among them, the dynamic contrast-enhanced magnetic resonance imaging (DCE-MRI) approach offers the unique advantage of combining high spatial resolution and tissue contrast with functional information [20]. Following the injection of a paramagnetic contrast agent (CA), it is possible to evaluate the tissue contrast enhancement produced by its extravasation through hyperpermeable tumor vessels and extrapolate pharmacokinetic parameters that provide information on vascular permeability and perfusion (*K* ^trans^), extracellular volume fraction (*v*_e_), and blood plasma volume fraction (*v*_p_) [21, 22]. DCE-MRI has proven to be a promising tool for the assessment of malignancy in different cancers, and the kinetic constants obtained can be exploited as biomarkers to assess tumor angiogenesis and response to antiangiogenic therapy [23–27].

We therefore hypothesized that quantitative permeability measurements might reveal characteristic vascularization properties of imatinib-sensitive and imatinib-resistant GIST tumors. This assumption is supported by studies that assessed perfusion in GIST tumors via contrast-enhanced ultrasonography and computed tomography [28, 29]. For this purpose, three mouse models of imatinib-sensitive and imatinib-resistant tumors in highly immunodeficient NOD scid gamma (NSG) mice were used. Functional MRI was exploited to characterize the tumor microenvironment in terms of vascularization and permeability by combining DCE-MRI with a new Gd-based blood pool contrast agent [30].

## Materials and methods

GIST cell-line culture and MRI image analysis are described in the Electronic supplementary material (ESM).

### Mice

Male 7-week-old NOD.Cg-Prkdc^scid^ Il2rg^tm1Wjl^/SzJ(NSG) mice with an average body weight of 30g were used. All animals were housed in sterile cages under laminar flow hoods in a temperature-controlled room with a 12-h light/12-h dark schedule and fed with autoclaved chow and water ad libitum. Mice were maintained at the animal facility of the Molecular Biotechnology and Health Sciences Department at the University of Turin and treated in accordance with the university’s ethical committee and European guidelines (Directive 2010/63) under protocol number 0081521.

Heterotopic GIST xenografts were generated by subcutaneous bilateral injection of the GIST882, GIST430, and GIST-T1 cell lines. For each cell line, *n* = 8 mice were bilaterally inoculated. GIST cells were suspended in 50 µL of phosphate-buffered saline (PBS) mixed with 50 µL of Matrigel™ Matrix (BD Pharmigen, Milan, Italy) at a density of 2 × 10^6^, 1 × 10^6^, and 2.5 × 10^4^ for GIST882, GIST430, and GIST-T1, respectively. Tumor growths were monitored weekly using a caliper, and tumor volume was calculated as [(length × width^2^)/2].

### In vivo imaging experiments

Anatomical T_2_-weighted (T_2w_) MRI acquisitions were performed weekly and tumor volumes were calculated by drawing a region of interest (ROI) on the image for both the tumors on the same mouse using the ITK-SNAP software (http://www.itksnap.org/pmwiki/pmwiki.php).

When the tumor volume reached 30–500 mm^3^, DCE-MRI experiments were performed. Mice were anesthetized by injecting a mixture of tiletamine/zolazepam (Zoletil 100; Virbac, Milan, Italy) 20 mg/kg and xylazine (Rompun; Bayer, Milan, Italy) 5 mg/kg, and a 27-gauge catheter was introduced into the tail vein for contrast agent (CA) injection. MR images were acquired with a 1 Tesla Aspect M2 MRI system (Aspect Magnet Technologies Ltd., Netanya, Israel). T_2w_ anatomical images were acquired to monitor tumor progression using a fast spin echo sequence (TR = 2500 s; TE = 44 ms; number of slices = 10; slice thickness = 1.5 mm; FOV = 40 mm; matrix = 152 × 160; NEX = 4; acquisition time = 3 m 20 s).

The dynamic contrast-enhanced magnetic resonance imaging protocol consisted of an axial T_1w_ 3D spoiled gradient echo sequence with the acquisition of three initial pre-contrast images followed by the injection of a gadolinium binding serum albumin CA (Gd-AAZTA-MADEC, CAGE Chemicals, Novara, Italy) through the catheter. After injection at a dose of 0.03 mmolGd/kg b.w., 37 dynamic post-contrast images were acquired with the following parameters: TR = 40; TE = 2.1 ms, flip angle = 60°, number of slices = 10, slice thickness = 1.5 mm, FOV = 40 mm, matrix = 128 × 128.

### Immunofluorescence staining

After MRI acquisition, 0.25 mg dextran–Texas Red 40 kD (Life Technologies, Monza, Italy) were intravenously injected into the mice to assess vessel leakage. Ten minutes later, the mice were sacrificed and their tumors were excised, embedded in optimal cutting temperature matrix compound (Tissue-Tek^®^ OCT™) for cryosection staining, and preserved at −80 °C. The Texas Red-conjugated dextran signal was amplified with polyclonal rabbit anti-Texas Red^®^ (Life Technologies). MVD was assessed by CD31 staining (monoclonal rat anti-CD31, BD Pharmigen). All secondary antibodies were purchased from Life Technologies (Alexa-Fluor^®^). Briefly, slices were incubated with 10% goat serum for 1 h at room temperature (RT) and then with primary antibodies (dilution 1:200) overnight at 4 °C. After washing in PBS–Tween 0.1%, the slices were incubated with secondary antibody (dilution 1:500) for 1 h at RT. Nuclei were stained with DAPI (Sigma–Aldrich, Milan, Italy) and the slices were rinsed with bidistilled water.

### Evaluation of MVD and MDD

Immunohistochemical assessment was performed using an ApoTome fluorescence microscope (ZEISS, Oberkochen, Germany). The degree of angiogenesis was determined by calculating the microvessel density (MVD) on CD31-positive slices and the extravasation of dextran as the mean dextran density (MDD). Microvessels were visualized as lumen-containing structures in which all single cells or clusters of cells were positive for CD31 immunoreaction. Staining for CD31 was visualized as a green signal (wavelength 488 nm, FITC green), whereas dextran extravasation emits in the red (wavelength 568 nm, Texas Red). The entire section was systematically scanned at ×100 magnification, ten fields were viewed at ×200 magnification, and images of CD31, dextran, and DAPI (wavelength 461 nm, blue) staining were taken. Two or more positive foci belonging to the same continuous vessel were counted as one microvessel, as described by Weidner et al. [31]. The MVD and MDD were manually counted and averaged over ten fields.

### Western blot samples and analysis

Cells from GIST 430-, 882- and T1-derived tumors were extracted with RIPA buffer (1% Triton X-100, 0.1% SDS, 1% sodium deoxycholate, 150 mM NaCl, 50 mM Tris–HCl pH 7, 0.4 mM Na_3_VO_4_, inhibitor mix). Cell lysates were centrifuged at 13,000×*g* for 10 min, and the supernatants were collected and assayed for protein concentration with the Bio-Rad protein assay method (Bio-Rad, Hercules, CA, USA). Total cell lysates were separated by SDS-PAGE under reducing conditions, transferred to nitrocellulose, and immunoblotted overnight with primary antibodies against vinculin (loading control), VEGFR2, and VEGFR3 at 4 °C. Mouse monoclonal antibody against vinculin was produced at the Molecular Biotechnology Center (MBC), while antibodies against VEGFR2 and VEGFR3 were purchased from Cell Signaling (Beverly, MA, USA). Blots were incubated with mouse or rabbit horseradish peroxidase conjugated secondary antibodies for 1 h at room temperature. ECL (Euroclone) was used to detect chemoluminescent signals. Protein band intensities were measured by a scanning densitometer (Quantity One; PDI Inc., Huntington, NY, USA).

### Statistical analysis

Statistical analysis of imaging data, microvessel counting, and western blot densitometry were performed using GraphPad Prism 5 software (GraphPad Inc., San Diego, CA, USA). All data are shown in this work as the mean ± SEM. One-way ANOVA analysis and Dunn’s multiple comparison tests were used to compare the functional mean MRI-based estimates obtained for the mice grafted with the GIST882, GIST-T1, and GIST430 cell lines.

One-way ANOVA analysis and Bonferroni multiple comparison tests were performed to evaluate statistical MVD and MDD differences among GIST tumors. The relationship between the ex vivo histological markers of vascularization (MVD and MDD) and the estimates obtained by DCE-MRI analysis (*K* ^trans^ and *v*_p_) were assessed with Pearson’s (parametric) rank correlation (*r*). For all tests, a *P* value of <0.05 was considered statistically significant.

## Results

### Generation of imatinib-sensitive and imatinib-resistant GIST models on NSG mice

**Fig. 1:**
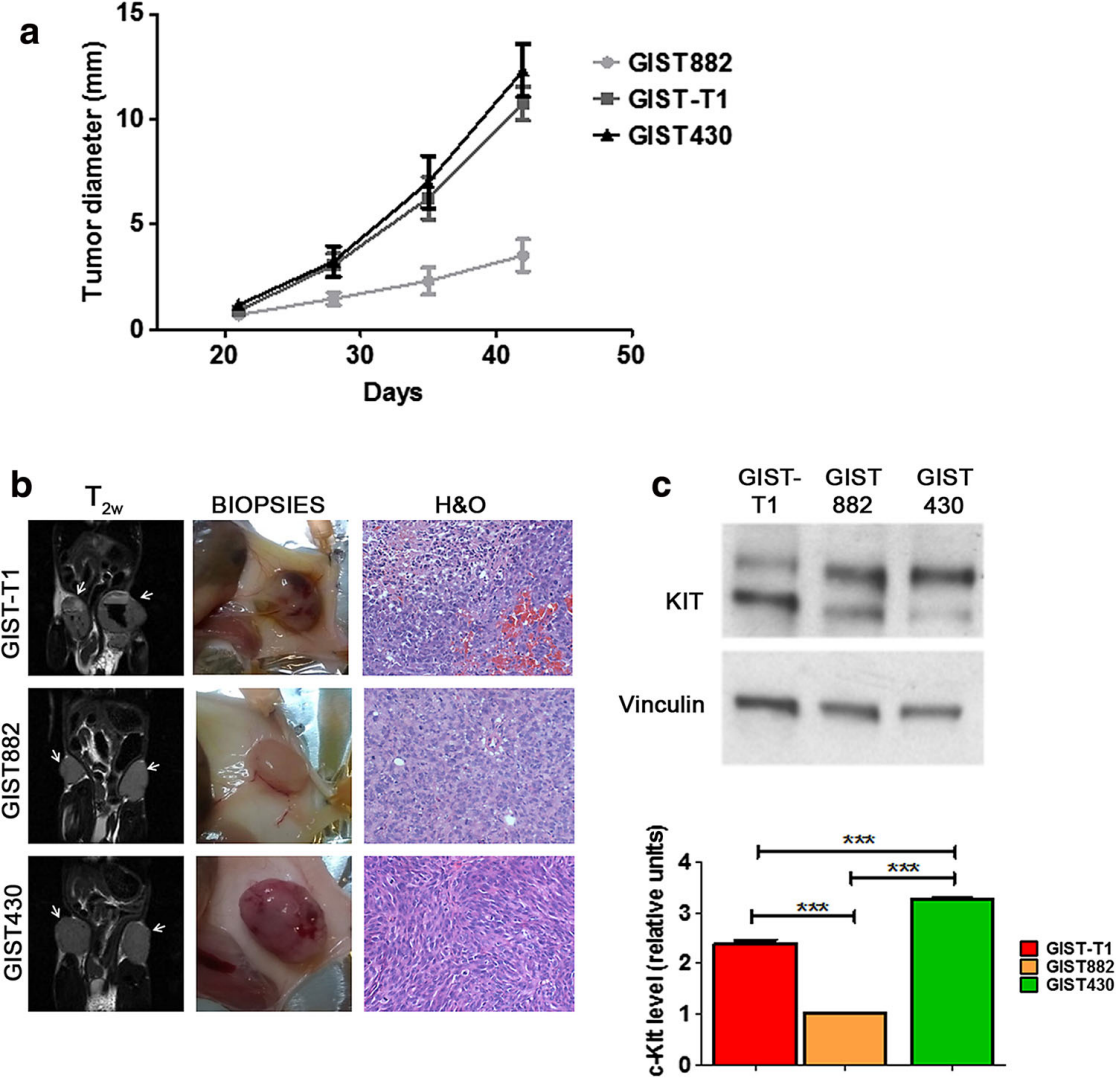
**a**–**c** Implementation of GIST-T1, GIST882, and GIST430 tumor models in NSG mice. **a** Curves indicating tumor diameter (mm) as measured with a caliper 14, 21, 28, 35, and 42 days after bilateral subcutaneous inoculation of the GIST cell lines (2.5 × 10^4^, 2.0 × 10^6^, and 1.0 × 10^6^ cells for GIST-T1, GIST882, and GIST430, respectively) into the NSG mice (*n* = 8 for each cell line). Tumor growth was detected at 21 days post-inoculation. GIST-T1 and GIST430 exhibited faster kinetic growth rates than GIST882. **b** Morphological characterization of the GIST tumors. *Left panel* shows coronal T_2w_ MRI images acquired with a 7 Tesla Bruker scanner of GIST-T1 (*top*), GIST882 (*middle*), and GIST430 (*bottom*) tumors (*arrowheads*). Hemorrhagic bleeding lesions are clearly present in the GIST-T1 tumors. *Middle panel* shows representative biopsies of excised GIST-T1 (*top*), GIST882 (*middle*), and GIST430 (*bottom*) tumors of mice sacrificed after MRI acquisition. The *arrowheads* indicate extensive bleeding in the GIST-T1 tumors. *Right panel* shows representative images of H&O staining of tumor sections from GIST-T1 (*top*), GIST882 (*middle*), and GIST430 (*bottom*) mice acquired using an optical microscope with a 20× objective. **c** Western blot analysis indicates that expression of the KIT receptor is increased in GIST430 compared to that in GIST-T1 and GIST882. Vinculin was provided as a loading control. Results of densitometric analysis of protein levels in at least three independent experiments are shown. Statistical analysis was performed using Student’s *t* test (*P < 0.05, **P < 0.01)

The GIST882, GIST-T1, and GIST430 cell lines were subcutaneously inoculated into NSG mice to generate imatinib-sensitive and imatinib-resistant GIST models. Solid tumors developed efficiently in all the animals considered for the study, and these tumors exhibited different kinetic growth rates depending on the inoculated GIST cell line (Fig. 1a). Palpable masses were typically detected from 15 to 18 days after inoculation. The GIST-T1 tumors displayed rapid growth rates, similar to those of the GIST430 tumors. Conversely, growth was much slower for the GIST882 tumors, reaching a maximum of 400 mm^3^ 45 days after inoculation. The mouse models exhibited different morphological features. Coronal T_2w_ MRI images highlighted highly hemorrhagic bleeding lesions in GIST-T1 tumors (Fig. 1b), partially similar to what was observed for the GIST430 tumors. Conversely, the GIST882 tumors exhibited dense and compact tissue with no signs of bleeding. Biopsies and histological H&O results showed substantial morphological differences among the GIST models, confirming MRI findings. Cellular and subcellular structures identified by H&O staining were in accordance with those previously reported elsewhere for all three GIST tumors [32, 33]. Different KIT expression levels among the GIST-T1, GIST882, and GIST430 tumors were noted in western blot analysis (Fig. 1c).

### DCE-MRI identifies differences in vascularization and permeability between imatinib-sensitive and imatinib-resistant GIST tumors

**Fig. 2:**
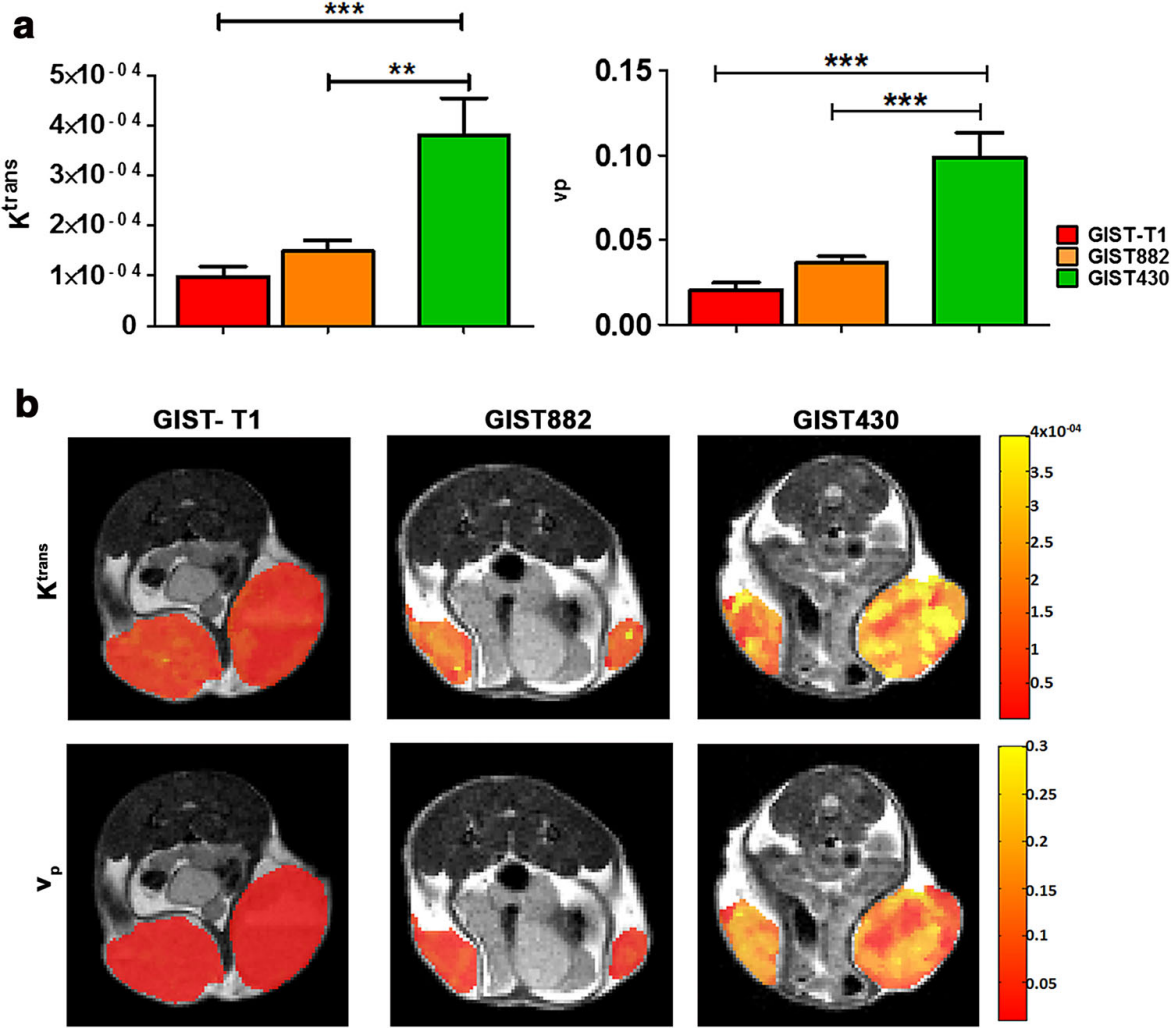
**a**–**b** Functional MRI estimates indicate higher vessel density and permeability in imatinib-resistant GIST430 tumors. **a** *Bar graphs* showing mean values of *K* ^trans^ (min^−1^, *left*) and *v*_p_ (*right*) obtained by DCE-MRI for imatinib-sensitive GIST-T1 (*black*) and GIST882 (*gray*) tumors as well as imatinib-resistant GIST430 tumors (*white*). GIST430 tumors display significantly increased *K* ^trans^ and *v*_p_ values than those for GIST-T1 and GIST882 tumors. Imatinib-sensitive tumors show comparable mean values for both *K* ^trans^ and *v*_p_. Values are shown as the mean ± SEM. Statistical significance: **P < 0.01; ***P < 0.001. **b** Representative parametric maps of *K* ^trans^ and *v*_p_ superimposed on related T_2w_ anatomical images. GIST-T1 (*left*), GIST882 (*middle*), and GIST430 (*right*) tumors show different values of *K* ^trans^ (*first line*) and *v*_p_ (*second line*). Parametric maps highlight increased values of the pharmacokinetic parameters in GIST430 in comparison to GIST-T1 and GIST882 tumors.

Functional MRI acquisitions were performed for GIST tumors with volumes in the range 30–500 mm^3^. Tumor microvessel permeability (*K* ^trans^) and plasmatic volume (*v*_p_) values were calculated by applying a two-compartment pharmacokinetic model to DCE-MR images following the administration of Gd-AAZTA-MADEC, a new blood-pool contrast agent (Fig. 2a). Imatinib-resistant GIST430 tumors exhibited significantly higher *K* ^trans^ mean values than imatinib-sensitive GIST882 and GIST-T1 tumors (38.1 ± 7.4×10^−5^ for GIST-430, 14.9 ± 2.2×10^−5^ for GIST882 and 9.8 ± 2.0×10^−5^ for GIST-T1, *P* = 0.0002, one-way ANOVA). A similar trend was observed for *v*_p_, that showed significantly higher values in the imatinib-resistant tumors than in the imatinib-sensitive ones (0.10 ± 0.01 for GIST430, 0.04 ± 0.004 for GIST882, and 0.02 ± 0.004 for GIST-T1; *P* < 0.0001, one-way ANOVA). Imatinib-sensitive tumors (GIST882 and GIST-T1) showed similar mean values for both estimates, without significant difference between the values. Representative parametric maps of *K* ^trans^ and *v*_p_ were overlaid on anatomical T_2_-weighted images; these are displayed in Fig. 2b. Qualitatively, these maps depict substantially increased *K* ^trans^ and *v*_p_ values in GIST430 tumors in comparison to those in GIST882 and GIST-T1 tumors.

### Ex vivo evaluation of tumor angiogenesis correlates with quantitative MRI parameters

**Fig. 3:**
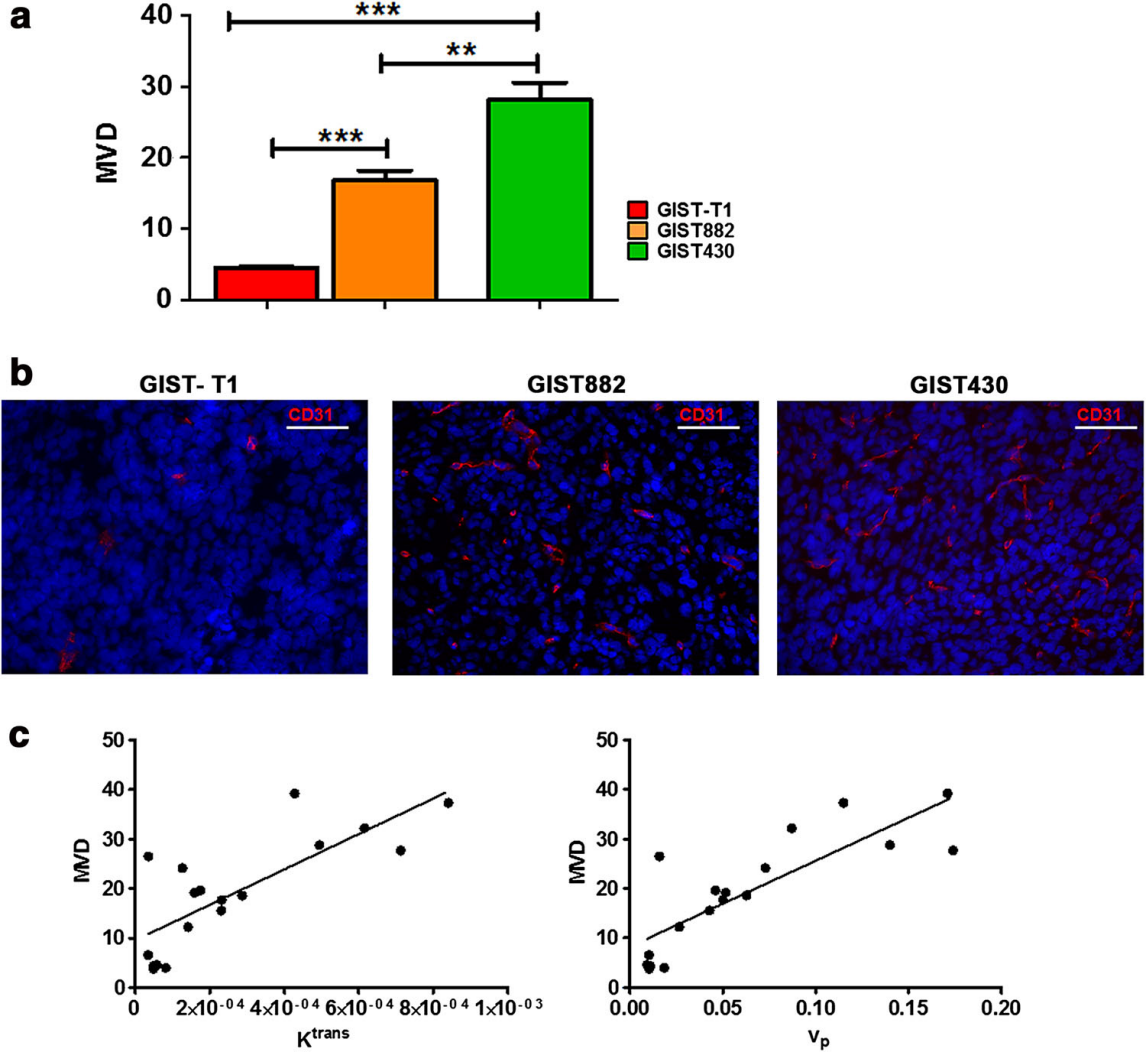
**a**–**c** Ex vivo analysis confirms the MRI findings by evaluating vascular density in GIST tumors. **a** Bar graph presents the microvessel density (MVD), calculated as number of vessels, for GIST-T1 (*black*), GIST882 (*gray*), and GIST430 (*white*) tumor sections. Vessels were immunostained for the endothelial marker CD31. GIST430 tumors show statistically significantly increased MVD in comparison to both GIST-T1 and GIST882 tumors. A significant difference was also observed between the MVD values of GIST-T1 and GIST882 tumors. Values are shown as the mean ± SEM. Statistical significance: *P < 0.05; ***P < 0.001. **b** Representative immunofluorescence staining for CD31 (*red*) in GIST-T1 (*left*), GIST882 (*middle*), and GIST430 (*right*) tumor sections. Nuclei were counterstained with Hoechst (*blue*). Images were acquired using a fluorescence microscope with a 20× objective. The GIST430 tumor section shows increased vessel density in comparison to the GIST-T1 and GIST882 tumor sections. **c** Correlation between MRI estimates *K* ^trans^ (*left*) or *v*_p_ (*right*) and histological MVD. Strong positive correlations were observed between both MRI estimates and their related MVD values (*v*_p_: *P* < 0.0001, *r* = 0.82; *K* ^trans^: *P* = 0.002, *r* = 0.78)

**Fig. 4:**
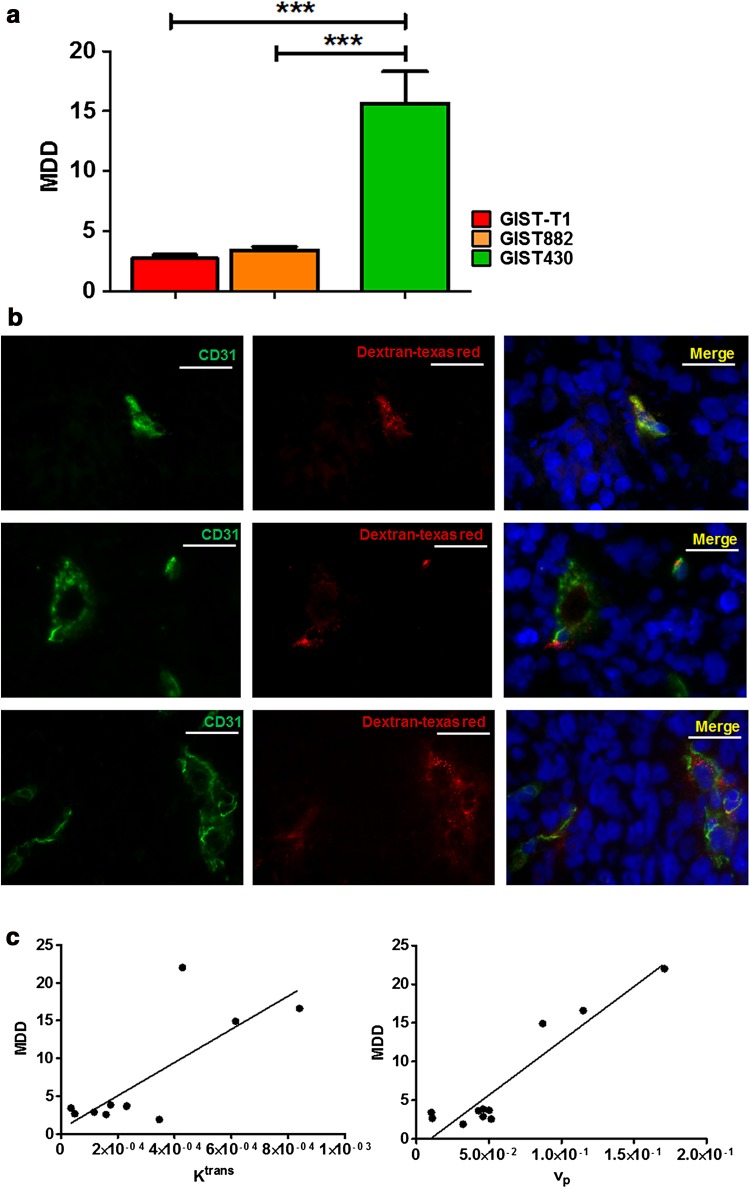
**a**–**c** Increased vessel permeability in GIST430 tumors is confirmed by dextran–Texas Red extravasation. **a** Bar graphs show the mean dextran density (MDD) calculated as the extravasation of dextran–Texas Red in positively CD31-immunostained vessels. GIST430 tumors display significantly higher dextran extravasation than GIST882 and GIST-T1 tumors. Values are shown as the mean ± SEM. Statistical significance: ***P < 0.001. Dextran–Texas Red was injected into the tail vein and mice were sacrificed 10 min later. **b** Representative images of CD31 (*green*) and dextran–Texas Red extravasation (*red*) in GIST-T1 (*first line*), GIST882 (*second line*), and GIST430 (*third line*) tumor sections. Images were obtained using a fluorescence microscope with a 40× objective. Merged images show counterstaining with Hoechst (*blue*). **c** Correlation between MRI estimates *K* ^trans^ (*left*) or *v*_p_ (*right*) and histological MDD. Strong positive correlations were observed between both MRI estimates and their related MDD values (*v*_p_: *P* = 0.0001, *r* = 0.94; *K* ^trans^: *P* = 0.006, *r* = 0.77)

Ex vivo staining for CD31 and dextran was performed to evaluate GIST vascularization and permeability, respectively. Quantitative analysis demonstrated that the GIST430 tumors were highly vascularized, with mean MVD = 31.9 ± 4.6 (Fig. 3a). Conversely, imatinib-sensitive GIST tumors displayed lower MVD values (MVD = 18.0 ± 0.9 for GIST 882, MVD = 4.9 ± 0.6 for GIST-T1). One-way ANOVA analysis showed significant differences in mean MVD between GIST430 and both GIST-T1 and GIST882 tumors (*P* = 0.0002). The representative immunofluorescence images shown in Fig. 3b of GIST-T1, GIST882, and GIST430 indicate different vascularization levels in GIST-T1, GIST882, and GIST430 tumor sections. Interestingly, MVD showed a strong positive correlation with both DCE-MRI estimates *v*_p_ (*P* < 0.0001, *r* = 0.82) and *K* ^trans^ (*P* = 0.002, *r* = 0.78) (Fig. 3c). The extravasation of dextran was assessed to evaluate functional vessel permeability ex vivo as the mean dextran density (MDD, Fig. 4a). MDD was more than threefold higher in GIST430 (15.7 ± 2.6) than in GIST882 and GIST-T1 tumors (2.7 ± 0.3 for GIST882 and 3.4 ± 0.3 for GIST-T1) with a statistically significant difference (*P* = 0.0003, Fig. 4a). Representative images of dextran–Texas Red extravasation and CD31-positive vessels are shown in Fig. 4b. Strong positive correlations were found between MDD and *K* ^trans^ (*P* = 0.006, *r* = 0.77) and between MDD and *v*_p_ (*P* = 0.0001, *r* = 0.94, Fig. 4c).

**Fig. 5:**
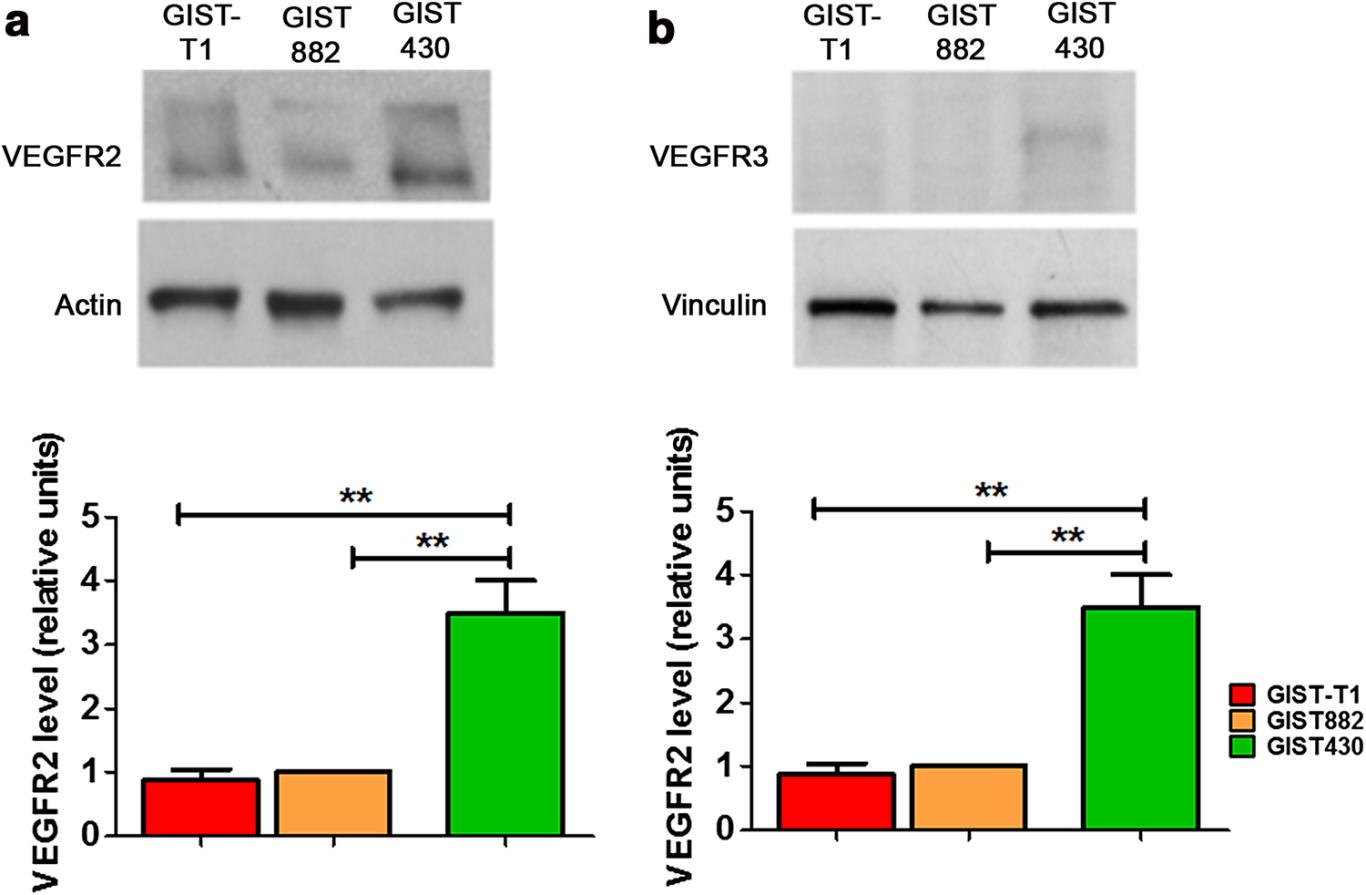
**a**–**b** Western blot analysis (**a**) and related bar graphs (**b**) of VEGFR2 and VEGFR3 in GIST-T1, GIST882, and GIST430 tumor samples. GIST430 tumors reveal significantly higher expression of both receptors in comparison to GIST-T1 and GIST882 tumors. Values are shown as the mean ± SEM. Statistical significance: *P < 0.05

Expression of endothelial receptors involved in tumor angiogenesis (VEGFR2) and lymphoangiogenesis (VEGFR3) as additional markers of tumor angiogenesis was investigated by western blot analysis in GIST tumors (Fig. 5a, b). Both VEGFR2 and VEGFR3 displayed more than threefold increases in expression in GIST430 compared to GIST-T1 and GIST882 tumors (*P* = 0.0015 for VEGFR2, *P* = 0.0007 for VEGFR3).

## Discussion

The aim of our work was to evaluate the ability of a functional MRI-based approach to highlight differences in tumor microenvironment properties related to imatinib resistance in GIST murine models. To this end, we investigated GIST tumor vascularization using a DCE-MRI approach. Our findings demonstrated that DCE-derived pharmacokinetic parameters can detect differences in plasmatic volume and permeability among the investigated GIST tumor cell lines. In particular, higher *K* ^trans^ and *v*_p_ values were measured for the imatinib-resistant tumors (GIST430) than for both the imatinib-sensitive tumors (GIST-T1 and GIST882). This study indicates that characterizing the tumor microenvironment and vasculature of GIST tumors using a functional MRI-based approach can allow imatinib-responsive tumors to be discriminated from imatinib-resistant tumors.

Angiogenesis is a fundamental step in the progression and metastasis of solid tumors. The clinical implications of angiogenesis and its prognostic significance have been reported in relation to several cancers, including lesions of the gastrointestinal tract [34–37]. Despite this, the risk of recurrence or the metastatic potential of a GIST is commonly predicted using the Fletcher classification system, which is mainly based on the evaluation of anatomic criteria. Only recently, several factors involved in the angiogenesis process were proposed as additional predictive biomarkers of the transition of a GIST from a benign to a malignant lesion. Ex vivo studies of GIST specimens have shown that MVD is closely related to VEGF expression and strongly associated with GIST prognosis. Those data indicated that angiogenesis and vascularization are associated with tumor grade, mitotic count, and higher risk of metastasis in GIST [15, 16]. Further confirming the key role that angiogenesis plays in GIST pathogenesis, we have shown in the present work, using a DCE-MRI approach, that imatinib-sensitive and imatinib-resistant tumors have different vascularization properties.

In particular, GIST430 tumors exhibit more than twofold higher *K* ^trans^ and *v*_p_ values compared to imatinib-sensitive tumors with a statistically significant difference. DCE-MRI results identified a more unstructured and deregulated vasculature in terms of blood flow and permeability (*K* ^trans^) and vascular density (*v*_p_), properties linked to the angiogenic process. Moreover, our in vivo functional findings were validated by histological quantifications of endothelial vessels (CD31) and permeability (dextran). Both of these parameters confirmed that GIST430 tumor sections presented higher vascularization and permeability compared to GIST-T1 and GIST882 tumors. These findings are in accordance with recent data from Imamura et al. [13], where higher expression of VEGF and increased MVD were observed in GIST tumors harboring a KIT mutation associated with resistance to imatinib. However, thus far, little is known about the association between angiogenesis and imatinib resistance in GIST. To the best of our knowledge, this is the first study to investigate tumor vascularization properties in several GIST-metastatic murine models and detect functional differences between imatinib-resistant and imatinib-sensitive tumors using an in vivo DCE-MRI approach. Interestingly, a significant positive correlation was found between *v*_p_ and MVD. The higher *v*_p_ values observed in the imatinib-resistant tumors indicate a larger vascular space; this result was confirmed by the observation of higher MVD values in GIST430, suggesting that *v*_p_ could be used as a marker of vessel density. In particular, considering the role of MVD in GIST prognosis, we hypothesize that *v*_p_ could be used to perform an in vivo assessment of GIST aggressiveness. GIST882 tumors showed significantly higher MVD values than GIST-T1 tumors, whereas no difference between the tumors was detected by DCE-MRI. We can explain this conflict by noting that *K* ^trans^ and *v*_p_ estimates assess only functional vessels whereas MVD assesses vessels regardless of their functionality. Consequently, DCE-MRI can detect poor perfusion properties of GIST882 tumors despite the presence of a relatively high vessel density.

Moreover, Yamashita et al. [38] observed intratumoral vessels in GIST patients with the worst prognosis in vivo using a contrast-enhanced ultrasound technique. The visualization of intratumoral vessels was correlated with VEGF expression, highlighting the relationship between angiogenesis and malignancy in GIST. Interestingly, we observed increased expression of VEGFR2 and VEGFR3 in GIST430 tumors in comparison to GIST882 and GIST-T1 tumors. VEGFR2 plays a well-known role in tumor angiogenesis formation and sprouting, whereas VEGFR3 is mainly involved in lymphoangiogenesis, which promotes and sustains tumor progression and angiogenesis and encourages metastases to spread through the surrounding lymphatic network. Several studies indicate that VEGFR3 is usually highly expressed in the most aggressive human cancers [39–41]. Our findings suggest that, in addition to VEGFR2, VEGFR3 expression may be relevant to imatinib-resistant GIST growth, and the direct targeting of these receptors could be a promising approach to treat nonresponding GIST tumors.

Antiangiogenic targeted therapies are considered key alternative treatment options in second- and third-line therapeutic regimens for GIST following imatinib resistance. Sunitinib is a multityrosine kinase inhibitor that is clinically approved for the treatment of imatinib-resistant GIST [42]. In addition to KIT and PDGFR, sunitinib targets the VEGFR1 and VEGFR2 receptors. Recently, Kim et al. [43] reported a pilot clinical study in which the effects of sunitinib treatment in GIST patients were quantitatively monitored by DCE-MRI. In particular, they observed significantly reduced *K* ^trans^ values with this treatment, which can be explained by the reduced wash-in rate caused by reduced vessel density. Their study, although limited to only a few patients, showed that the DCE-MRI technique can detect vascular functional changes in treated GIST, and that this can be exploited as a valid alternative to conventional treatment assessment approaches. In addition, perfusion imaging studies based on contrast-enhanced computed tomography demonstrated lower perfusion values in good responders, whereas poor responders showed significantly lower perfusion values [44, 45]. Our study clearly demonstrated in GIST mouse models that imatinib-resistant tumors exhibit higher *K* ^trans^ and *v*_p_ values than imatinib-sensitive tumors. Therefore, changes in these functional properties can be noninvasively monitored by DCE-MRI to assess the efficacy of antiangiogenic treatments such as sunitinib or other specific drugs that are currently in phase II or III clinical trials [46].

Several findings suggest that evaluations of tumor neovascularity and associated permeability changes can be improved by using macromolecular Gd-based CA adducts, which can accumulate within the tumor owing to enhanced permeability and retention effects [47]. The main advantages of these adducts are that they allow better assessment of tumor vascularization properties than small molecular weight CAs and they show higher contrast efficiency at low-to-intermediate magnetic fields (0.5–1.5 T) [48–50]. Our results, obtained using a new Gd-based blood pool CA [30], demonstrate that accurate characterization of GIST tumor microvascular properties is feasible at low magnetic fields, hence facilitating translational purposes at clinicl level.

This study has some limitations. First, only GIST cell lines that are sensitive or resistant to imatinib were investigated. Further studies are needed to explore GIST cell lines that are sensitive or resistant to other TK inhibitors (e.g., sunitinib or regorafenib). Moreover, only a small number of GIST cell lines were investigated: mice were inoculated with two imatinib-sensitive (GIST-T1 and GIST882) cell lines and one imatinib-resistant (GIST430) cell line. Further evaluations of additional GIST cell lines and patient-derived tumors could extend the applicability of the proposed MRI approach for characterizing GIST tumors, including the assessment of novel TK inhibitors [51, 52]. However, GIST-T1, GIST882, and GIST430 cells are the most commonly used and well-characterized GIST cell lines, and are usually considered to be representative of imatinib-sensitive (GIST-T1 and GIST882) and imatinib-resistant (GIST430) tumors. An additional limitation of this study is that clinical trials are needed to confirm that DCE-MRI is a valuable tool for assessing GIST tumor treatment response.

In conclusion, our work highlights the important role that functional MRI approaches can play in detecting functional differences between imatinib-sensitive and imatinib-resistant GIST tumors. In particular, differences in microvessel permeability and density among GIST tumors are highlighted by our DCE-MRI approach. Imatinib-resistant tumors exhibit increased *K* ^trans^ and *v*_p_ values compared to imatinib-sensitive ones, as confirmed by our ex vivo quantifications of MVD and MDD in GIST430 tumor sections. In addition, a strong positive correlation was observed between MRI and histological estimates. The current study suggests that the assessment of angiogenesis could be considered a promising new biomarker of response to imatinib treatment. Thus, DCE-MRI warrants more attention at the clinical level for the noninvasive assessment of treatment response through the evaluation of tumor vascularization properties. In view of the few therapeutic options that are currently available for imatinib-resistant GIST patients, angiogenesis targeting may be an effective therapeutic strategy for such patients, and functional MRI approaches may provide alternatives to conventional imaging modalities for the early detection of tumor response.

## Electronic supplementary material

Below is the link to the electronic supplementary material.

Supplementary material 1 (DOCX 20 kb)
