# Supplementary material for: Assessing tumor vascularization as a potential biomarker of imatinib resistance in gastrointestinal stromal tumors by dynamic contrast-enhanced magnetic resonance imaging
Source: Gastric Cancer. 2016 Dec 19;20(4):629–39. doi: 10.1007/s10120-016-0672-7 (PMC5486478; doi:10.1007/s10120-016-0672-7)
Supplement: Supplementary file 1 — Supplementary material 1 (DOCX 20 kb) [file 10120_2016_672_MOESM1_ESM.docx]

**Supplementary Information**

**TITOLO**

Lorena Consolino^1,2^, Dario Livio Longo^1,3^, Marianna Sciortino^1^,Walter Dastrù^1^, Sara Cabodi^1^, Giovenzana Giovanni Battista^2^, Silvio Aime^1^

^1^ Department of Molecular Biotechnology and Health Sciences, University of Torino, Via Nizza 52, 10126 Torino, Italy

^2^Cage Chemicals –Via Bovio 6, 28100, Novara, Italy

^3^Istituto di Biostrutture e Bioimmagini, (CNR) c/o Molecular Biotechnologies Center, Via Nizza 52, 10126, Torino, Italy

**METHODS**

**Cell lines and GIST xenografts**

GIST-T1 and GIST882 were established from human, untreated, metastatic GISTs. GIST-T1 contains a deletion in *KIT* exon 11, whereas GIST882 shows a missense mutation in *KIT* exon 13, encoding a K642 mutant *KIT* oncoprotein.^1,2^ GIST430 were established from GIST that had progressed, after initial clinical response, during imatinib therapy. GIST430 has a heterozygous primary KIT exon 11 in-frame deletion, followed by a heterozygous secondary exon 13 missense mutation (V654A).^3^ GIST882 and 430 were respectively cultured in RPMI and IMDM medium, both containing 15% Fetal Bovine Serum (FBS), 1% PenStrep, 100xGentamycin and 1% L-Glutamine. GIST -T1 were cultured in DMEM Glutamax medium containing 10% FBS and 1% PenStrep. Cells were maintained at 37°C in a humidified atmosphere of 5% CO_2_. All products were purchased from Life Technologies (Monza, Italy). GIST cell were provided by the European Community’s Seventh Framework Program (FP7 Mitigate project).

Heterotopic GIST xenografts were generated by subcutaneous bilateral injection of GIST882, GIST430 and GIST-T1. For each cell line, n=8 mice have been inoculated. GIST cells were suspended in 50 µl of Phosphate Buffered saline (PBS) mixed with 50 µl of Matrigel™ Matrix (BD Pharmigen, Milano, Italy) at a density of 2x10^6^, 1x10^6^ and 2,5x10^4^ for GIST882, GIST430 and GIST-T1, respectively. Tumor growths were weekly monitored over time by using a caliper and tumor volumes calculated by [(length x width^2^)/2].

**MRI images analysis**

MITK (<http://www.mitk.org/MITK>), ITK and VTK libraries were implemented for analyzing DCE-MRI images by using an in-house developed software in C++ code for the quantification of pharmaco-kinetic parameters and Matlab (MathWorks, Natick, MA).

For motion correction on VFA and DCE images, the correspondent anatomical T_2w_ images was taken as reference and a rigid-ITK co-registration was applied. Pre-contrast T_1_ values have been obtained using a variable flip angle fast gradient echo technique.[^4^](#_ENREF_38)Signal intensity (SI) curves were converted into longitudinal relaxation rate R_1_ (1/T_1_) and dynamic post-contrast T_1_ relaxation calculated by assuming a linear relationship between R_1_ and CA concentration according to the following equation:

$$C_{t}\left( t \right)=\frac{\frac{1}{T_{1}}-\frac{1}{T_{10}}}{r_{1p}}$$

Where 1/T_10_ indicates the pre contrast longitudinal relaxation rate, 1/T_1_ is the post contrast longitudinal relaxation rate and r_1p_ is the longitudinal relaxivity of the CA that is assumed to be equal to the value (40 mM^-1^ s^-1^) measured in blood serum. Arterial input function (AIF) has been individually measured by applying the extended Tofts’ model.^5^This model assumes a bidirectional exchange between the intravascular and the extravascular extracellular space (EES) compartments. The kinetic parameters (K^trans^, v_p_ and k_ep_) were extracted on on a voxel-by-voxel basis. For this purpose, the concentration curve in the tissue C_t_(t) has been fitted against the solution of the differential equation:

$$C_{t}\left( t \right)=v_{p}C_{p}\left( t \right)+K^{trans}\left[ C_{p}\left( t \right)\bigotimes e^{-k_{ep}(t)} \right]$$

where C_t_(t) is the CA concentration in the tissue at time *t*, v_p_ is the fractional blood plasma volume, C_p_(t) is the CA blood plasma concentration at time *t* (AIF), K^trans^ is the volume transfer constant between the intravascular and the EES (K^trans^=k_ep_V_e_), k_ep_ is the rate constant from EES to blood plasma and ⊗is the convolution operator.The software automatically determinedthe AIF and the injection time by using a three-dimensional region growing algorithm with an artery seed point automatically determined from the maximum increase of signal in the dynamic series. The same operator manually drawn a region of interest (ROI) for both tumors encompassing all the tissue volume taking the T_2w_ images as reference. K^trans^ and v_p_ mean values were extracted superimposing K^trans^ and v_p_ maps to manual drawn ROI of tumors (in house developed MATLAB scripts, The MathWorks, Inc., Natick, MA, USA).

**References**

1. Tuveson DA, Willis NA, Jacks T, Griffin JD, Singer S, Fletcher CD, Fletcher JA, Demetri GD. STI571 inactivation of the gastrointestinal stromal tumor c-KIT oncoprotein: biological and clinical implications. Oncogene 2001;20:5054-8.

2. Taguchi T, Sonobe H, Toyonaga S, et al. Conventional and molecular cytogenetic characterization of a new human cell line, GIST-T1, established from gastrointestinal stromal tumor. Lab Invest 2002;82:663-5.

3. Bauer S, Yu LK, Demetri GD, Fletcher JA. Heat shock protein 90 inhibition in imatinib-resistant gastrointestinal stromal tumor. Cancer Res 2006;66:9153-61.

4. Brookes JA, Redpath TW, Gilbert FJ, Murray AD, Staff RT. Accuracy of T1 measurement in dynamic contrast-enhanced breast MRI using two- and three-dimensional variable flip angle fast low-angle shot. Jmri-Journal of Magnetic Resonance Imaging 1999;9:163-171.

5. Tofts PS. Modeling tracer kinetics in dynamic Gd-DTPA MR imaging. J Magn Reson Imaging 1997;7:91-101.
